# Aging Characteristics of ZSM-5 Zeolite on Low-Frequency Acoustic Applications

**DOI:** 10.3390/nano15090639

**Published:** 2025-04-23

**Authors:** Mingbo Guo, Yijun Wang, Lei Zhang, Junran Lu, Chang Gong, Wanning Zhang, Yuxi Fang, Xinyuan Zhu, Shunai Che

**Affiliations:** 1 State Key Laboratory of Synergistic Chem-Bio Synthesis, School of Chemistry and Chemical Engineering, Frontiers Science Center for Transformative Molecules, Shanghai Key Laboratory for Molecular Engineering of Chiral Drugs, Shanghai Jiao Tong University, Shanghai 200240, China; mbguo_jeff@sjtu.edu.cn (M.G.); zhangwanning@sjtu.edu.cn (W.Z.); sjtu15901600323@sjtu.edu.cn (Y.F.); 2 SSI New Material (Zhenjiang) Co., Ltd., 7 Songlin Mountain Road, Zhenjiang 212006, China; yj.wang@n-bass.com (Y.W.); steven.zhang@n-bass.com (L.Z.); jr.lu@n-bass.com (J.L.); chang.gong@n-bass.com (C.G.)

**Keywords:** ZSM-5, acoustic enhancement materials, resonance frequency, microspeaker, aging

## Abstract

Zeolite is increasingly recognized for its enhancement of low-frequency acoustic performance in microspeakers. The aging characteristics of zeolite have been regarded as the critical factor for the commercial viability of mobile phones products, but the mechanism remains ambiguous. Here, the low-frequency acoustic performance of hierarchically structured ZSM-5 was investigated through aging with water and acetic acid (AA). It was discovered that water vapor augmented the resonance offset as it enhanced the structure of the zeolite, resulting in a lower water content. The resonance offset of ZSM-5 significantly decreased after the adsorption of AA vapor, as excessive AA was adsorbed through both physical and chemical adsorption, causing partial destruction of supermicropore and mesopores. The performance of ZMS-5 stored with vapor of AA and water mixture did not significantly deteriorate, indicating that water effectively protected the pores of zeolite to prevent excessive adsorption of AA. This was attributed to the fact that water was adsorbed by Brønsted acid sites of ZSM-5 more preferentially than AA, thereby avoiding excessive adsorption of AA.

## 1. Introduction

Microspeakers [1] are acoustic transducers that convert electrical power into mechanical energy, driving the diaphragm to vibrate and generate sound waves, which are widely used in mobile intelligence devices such as cell phones [2], laptops [3], augmented reality (AR) and virtual reality (VR) [4], etc. The size of the back volume in the loudspeaker has been recognized as a significant factor influencing low-frequency performance, with a larger back volume generally being more advantageous [5]. However, the incorporation of a large back volume into modern mobile phones is impractical due to their increasingly sleek and compact designs. The demand for high-quality acoustic performance conflicts with the shrinking back volume. Shuji Saiki [6] reported acoustic enhancement materials (AEMs) that were obtained by adding binders to porous materials such as activated carbon, zeolite, silica, alumina, zirconia, magnesia, etc. The speaker system filled with AEMs reproduced low-frequency sound performance even when using a smaller back volume. Activated carbon [7], zeolite [8], and mesopore silica [9] have been reported as AEMs which can improve low-frequency acoustic performance of microspeakers. It is assumed that porous materials would adsorb a portion of the compressed air in the back volume when subjected to vibration by the diaphragm. The lack of air molecules facilitates diaphragm vibration and the compliance of the loudspeaker is improved, thereby improving acoustic performance [7]. The enhanced acoustic performance of microspeakers is attributed to air adsorption rather than sound absorption. This distinguishes AEMs completely from sound-absorbing materials (SAMs). The air adsorption capacity is regarded as accountable for the enhanced acoustic performance [8].

However, for a commercial product, several issues need to be addressed. The working environment of microspeakers differs significantly from that of chemical reactors. Specifically, moisture and acid gas in the air are continuously adsorbed and desorbed by AEMs, which may compromise the integrity of their pore structure due to the inherent adsorption properties of porous materials. Even trace amounts of acidic gas in the air can lead to the accumulation of adsorbates, potentially clogging the pores and causing degradation of AEMs. The mechanism is analogous to the deactivation of zeolite catalysts caused by coke deposits within their pore structures [10]. Therefore, research on the aging behavior of AEMs is crucial for their successful commercialization.

The resistance to moisture and acetic acid (AA) is a critical parameter for AEMs [11]. Moisture in the air is inevitably adsorbed by AEMs during operation in microspeakers. Additionally, significant concentrations of AA have also been found in small closed spaces [12]. The adsorption of AA on zeolite is complex; it is not merely a physical adsorption behavior. AA can be converted to isobutene catalyzed by Beta zeolite [13] and to acetone on the zeolite H-ZSM-5 with Lewis/Brønsted-based acid sites [14]. To date, the adsorption behavior of moisture and AA on zeolite-based AEMs has not been reported, and the underlying mechanisms remain unclear. It has been reported that zeolites with monovalent metal cations exhibit a higher nitrogen adsorption capacity [15] and K-type ZSM-5 has a lower charge density compared to Na-type ZSM-5 [16]. Water or other polar molecules adsorb on the surface of zeolite via dipole–field interaction, and a low charge density leads to a low adsorption of polar molecules [17]. Here, the aging characteristics of AEMs were investigated by hierarchical structured ZSM-5 with potassium ion exchange. The co-existence of water and AA was employed to simulate the aging behavior of ZSM-5. The acoustic enhancement performance was evaluated by monitoring the changes in resonance frequency (*f*_0_) and sound pressure level (SPL@500Hz) read from an impedance curve and a frequency response curve. In situ diffuse reflectance infrared Fourier-transformed spectroscopy (DRIFTS) was utilized to investigate the adsorption–desorption behavior of water and AA. Nitrogen isotherms were used as an important protocol to investigate the change in the pore structure of zeolite after the aging tests. This research provides valuable insights into the aging characteristic of zeolite-based AEMs.

## 2. Materials and Methods

### 2.1. Experimental Section

#### 2.1.1. Materials

Colloidal silica (30 wt%) was procured from Shandong Peak-tech New Material Co., Ltd. (Linyi, China). Aluminum sulfate octadecahydrate (Al_2_(SO_4_)_3_•18H_2_O), sodium hydroxide (NaOH), Tetrapropylammonium bromide (TPABr), potassium chloride (KCl) and AA were obtained from Aladdin Biochemical Technology Co., Ltd. (Shanghai, China), and were of analytical grade. Deionized water with a conductivity of <5 μS/cm was prepared by the lab water purification system.

#### 2.1.2. Synthesis of Zeolite

Preparation of Na-type ZSM-5 (Na-Z5): The zeolite sample was synthesized using a conventional hydrothermal method. Briefly, 3.33 g of Al_2_(SO_4_)_3_•18H_2_O was added into 520.0 g of deionized water and stirred until complete dissolution was achieved. Subsequently, 24.0 g of NaOH and 320.0 g of tetrapropylammonium bromide (TPABr) were gradually added to the solution while continuously stirring until full dissolution occurred. The synthesis molar composition was SiO_2_:Al_2_O_3_:TPABr:NaOH:H_2_O = 4:0.005:1.2:0.6:60, corresponding to a silica-to-alumina molar ratio (SAR) of 800. Finally, 800.0 g of colloidal silica was incorporated at an extremely slow pace to prevent white flocculation. An additional 30 min stirring was conducted until a uniformly milk-white gel was achieved. The gel was transferred into a 2 L autoclave lined with stainless steel and properly sealed. The autoclave was heated to 80 °C at a rate of 1 °C/min and maintained at 80 °C for 8 h. Subsequently, it was heated to 150 °C with a heating rate of 1 °C/min and held at 150 °C for 24 h.

After crystallization, the gel was retrieved when the autoclave cooled to below 50 °C and underwent a washing process subsequently. The mother solution was removed by a centrifuge initially, and the residue cake was rinsed with deionized water at a temperature ranging from 50 to 60 °C until the conductivity of the washing water was lower than 100 μS/cm. The washed cake was dried at 120 °C overnight and then subjected to a calcination procedure in a muffle furnace after being crushed. The muffle furnace was heated to 120 °C at a rate of 5 °C/min and remained at that temperature for 120 min. Subsequently, it was heated to 300 °C with a heating rate of 2 °C/min and held at this temperature for 60 min. Finally, the furnace was heated to 550 °C at a rate of 2 °C/min and maintained for 240 min; the powder after calcination was labeled as Na-Z5.

Preparation of K-type ZSM-5 (K-Z5): An ion exchange process was employed for Na-Z5. Firstly, 100 g of the calcined Na-Z5 powder was blended with 1000 g of deionized water, and subsequently, 15 g of potassium chloride was added into the mixture. The mixture was heated to 80 °C and kept for 4 h while being stirred. A protocol similar to the one mentioned above, encompassing washing, drying, pulverizing and calcination, was adopted after the reaction. The final sample after calcination was designated as K-Z5.

#### 2.1.3. Characterization

The mole ratio of silica to alumina (SAR) of the samples was measured using a ZSX Primus III+ from Rigaku (Shanghai, China) operating at tube voltage of 50 kV and tube current of 50 mA employing semi-quantitative analysis.

X-ray diffraction (XRD) patterns were measured by a PANalytical X’Pert^3^ instrument from Malvern (Shanghai, China) with Cu Kα radiation (45 kV, 40 mA, λ = 0.154056 nm) at a scanning rate of 8°/min.

Nitrogen adsorption/desorption isotherms were measured at 77 K by means of the BELSORP-Max Nitrogen Physical Adsorption Instrument manufactured by Microtrac (Osaka, Japan). Before the measurement, the sample underwent a pretreatment involving degassing at 300 °C for 90 min. The surface area was calculated through the Brunauer–Emmett–Teller (BET) method, and pore size distribution was determined by nonlocal density functional theory (NLDFT) using adsorption isotherms.

Thermogravimetric analysis (TGA) was performed by PE TGA 4000 from PerkinElmer (Shanghai, China) and the parameters were as follows: heating temperature from 25 to 800 °C (heating rate of 10 °C/min) with a nitrogen flow of 20 mL/min.

Attenuated total reflectance–infrared spectroscopy (ATR-IR) spectra were recorded with a Nicolet iS5 from Thermo Fisher Scientific (Shanghai, China) with resolution of 0.8 cm^−1^ by 16 scans.

The morphology of zeolite samples was observed with a ZEISS Sigma 300 field emission scanning electron microscope (FE-SEM) from ZEISS (Shanghai, China).

The internal cross-sectional morphology of zeolite crystals was observed using a FEI Talos F200X G2 transmission electron microscope (TEM) from Thermo Fisher Scientific (Shanghai, China) with a 100 nm ultrathin section cut by Leica UC7 using a diamond blade.

In situ diffuse reflectance infrared Fourier-transformed spectroscopy (DRIFTS) was performed by IS 50 from Thermo Fisher Scientific (Shanghai, China); the measurement of all spectra was carried out between 600 and 4000 cm^−1^ with a spectral resolution of 4 cm^−1^ and the reported spectra were obtained by an average of 32 scans. Prior to the test, the samples in the chamber were degassed at 80 °C for 1 h, and then purged with nitrogen for 10 min.

#### 2.1.4. Acoustic Performance Measurement

The impedance curve and the frequency response curve were measured in accordance with “IEC 60268-5 Sound system equipment—Part 5: loudspeaker” [18]. To guarantee data consistency, both curves were tested at 23 ± 2 °C and within a relative humidity range of 40~70% in an anechoic chamber using a microspeaker with a back volume of 0.42 cm^3^. The impedance curve and the frequency response curve were measured by a soundcheck purchased from Listen.

**Figure 1:**
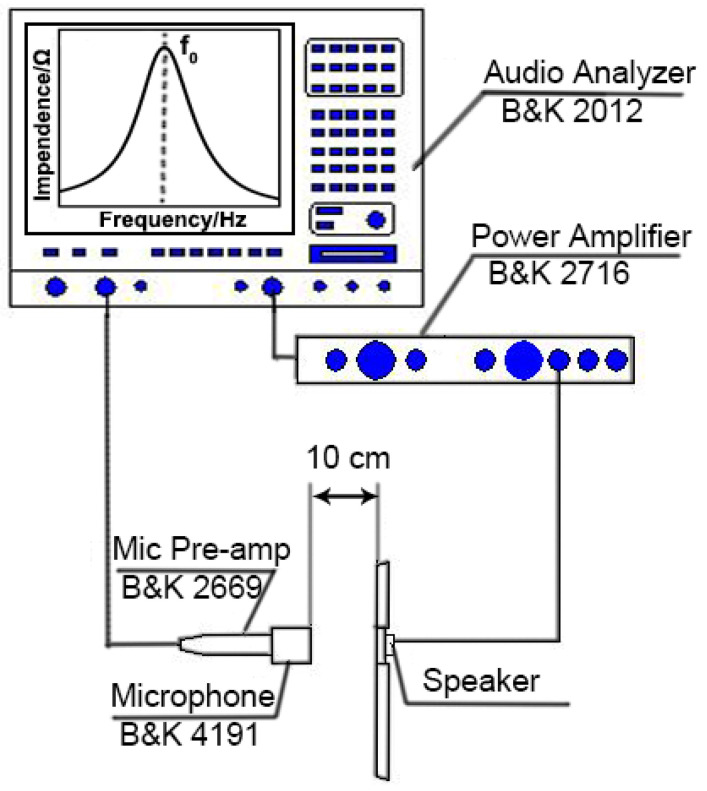
Schematic diagram of the measuring apparatus for measuring the impedance curve (*f*_0_) and frequency response curve.

Figure 1 shows the schematic diagram of the measuring device used for measuring the impedance curve and the frequency response curve. When sinusoidal electrical signals were applied to the microspeaker, the device’s inherent impedance produced a corresponding voltage response. These analog signals were then conditioned through a power amplification stage before being routed to the audio analyzer for impedance curve generation through spectral analysis. Concurrently, the microspeaker transduced input electrical energy into corresponding acoustic emissions through electromechanical coupling. The resultant pressure waves were captured by a reference-grade measurement microphone and generating transducer-specific electrical output, which were amplified via the Mic Pre-amp, and subjected to precision analysis by the Audio Analyzer to obtain the frequency response curve. The curves were initially measured without any material filled into the back volume as a reference, which was termed “Empty cavity”. The acoustic performance of the samples was evaluated by the offset of resonance frequency (Δ*f*_0_) and the increase in the SPL@500Hz reading from the two curves in comparison to “Empty cavity” when 0.060 ± 0.001 g samples filled in the back volume. Resonance frequency (*f*_0_) was the X-axis value of the first peak in the impedance curve (Figure 1), and SPL@500Hz was the SPL value at 500 Hz obtained from the frequency response curve. The *f*_01_ and SPL@500Hz_01_ were measured with an empty back volume initially, whereas *f*_02_ and SPL@500Hz_02_ were measured with AEMs filled in the back volumes. The acoustic enhancement performance of the AEMs was quantitatively assessed using Δ*f*_0_ and ΔSPL@500Hz, which were calculated based on the following formulas.

$$∆f_{0}=f_{01}-f_{02}\;∆SPL@500Hz={SPL@500Hz}_{02}-{SPL@500Hz}_{01}$$

#### 2.1.5. Aging Experiments

Aging with H_2_O vapor: 0.4 g of K-Z5 was placed in a 250 mL glass bottle with 100 g of water at the bottom (Figure S1), and the bottle was placed in an oven at 85 °C for 4 h. Subsequently, the sample was taken out and cooled at room temperature for 90 min, and was denoted as K-Z5-H_2_O.

Aging with AA vapor: 0.4 g of K-Z5 was placed in a 250 mL glass bottle with 100 g of AA at the bottom. The sealed bottle was then placed in an oven at 85 °C for 4 h. Subsequently, the sample was taken out and left at room temperature for 90 min, and was labeled as K-Z5-AA.

Aging with vapor of AA and H_2_O mixture: 0.4 g of K-Z5 was placed in a 250 mL glass bottle with 100 g of 1 M AA aqueous solution at the bottom, and the bottle was placed in an oven at 85 °C for 4 h. Subsequently, it was taken out and cooled at room temperature for 90 min, and the sample was labeled as K-Z5-H_2_O&AA.

## 3. Results and Discussion

### 3.1. Characterization Analysis

The SAR was determined by XRF, and the result is presented in Table 1. The SAR of K-Z5, K-Z5-H_2_O, K-Z5-AA and K-Z5-H_2_O&AA was 608, 613, 646 and 617, respectively. The SAR of K-Z5-H_2_O and K-Z5-H_2_O&AA was slightly higher than that of K-Z5. However, the difference was negligible. The SAR of K-Z5-AA was higher than that of K-Z5, which could be attributed to dealumination, a process that commonly occurs when zeolites are treated with acid [19].

**Figure 2:**
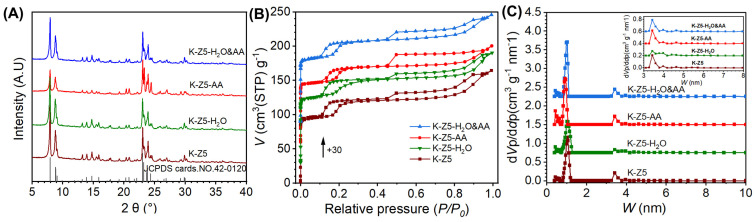
Structural characterizations of K-Z5, K-Z5-H_2_O, K-Z5-AA and K-Z5-H_2_O&AA. (**A**) XRD patterns. (**B**) N_2_ adsorption–desorption isotherms. (**C**) Pore size distribution.

The XRD patterns of K-Z5, K-Z5-H_2_O, K-Z5-AA and K-Z5-H_2_O&AA are presented in Figure 2A. The XRD pattern of K-Z5 corresponds to the MFI topology structure, exhibiting five characteristic peaks at 2*θ* = 7.9, 8.9, 23.0, 23.9, and 24.4° [20], and no impurity was identified based on the standard card (JCPDS cards No. 42-0120). The doublet peaks at 24.4° and 29.9° indicated that the crystal system of K-Z5 exhibits monoclinic symmetry, a feature commonly observed in ZSM-5 zeolites with high Si/Al ratios [21]. The XRD patterns of K-Z5-H_2_O and K-Z5-H_2_O&AA are analogous to K-Z5, with nearly identical intensities for the characteristic ZSM-5 peaks. In contrast, the XRD pattern of K-Z5-AA differs slightly from that of K-Z5. The peaks at 24.4° and 29.9° appear as singlets, indicating an orthorhombic crystal system. Additionally, the intensities of the peaks at 7.9° and 8.9° are reduced. The diffraction peak at 7.9° represents the (101) crystal plane, while the peak at 8.7° corresponds to the (020) crystal plane and the straight channel of the MFI structure [22]. Figure S4 illustrates the pore structure of the ZSM-5 zeolite. It is well established that the ZSM-5 zeolite (MFI topology) features a two-dimensional sinusoidal channel system (5.1 Å × 5.5 Å) and a one-dimensional straight channel system (5.3 Å × 5.6 Å), both comprising a dual 10-membered ring (10-MR) microporous framework. The straight channels extend along the b-axis, exposing (010) facets, with (020) facets being parallel to them. The sinusoidal channels run parallel to the a-axis, exposing (100) and (101) facets. [23] The lower intensity of the two peaks of K-Z5-AA suggests that the pores and channels adsorbed a considerable amount of AA after the aging experiment.

Nitrogen adsorption–desorption isotherms (Figure 2B) of all four samples were similar, exhibiting a combination of type I and type IV, which indicates the coexistence of micropores and mesopores [24]. Two distinct hysteresis loops were observed in the isotherms at partial pressures (*P*/*P*_0_) within the ranges of 0.1 to 0.4 and 0.4 to 1.0, respectively. The hysteresis loop at *P*/*P*_0_ = 0.4 to 1.0 was typically witnessed in hierarchical ZSM-5, suggesting the existence of abundant mesopores within the crystals and capillary condensation taking place in these mesopores [25]. The hysteresis loop at *P*/*P*_0_ = 0.1 to 0.4 commonly known as the “low pressure hysteresis loop” and was related to a fluid-to-crystalline phase transition of the adsorbed nitrogen [26].

The NLDFT pore size distribution (Figure 2C) revealed that, in addition to the conventional micropores with a pore size of approximately 0.5 nm characteristic of ZSM-5 [27], K-Z5 also exhibited supermicropores with a size of approximately 1 nm and mesopores with a size of approximately 3.5 nm. The size of mesopores increased after aging with water, as evidenced by the broader size distribution of mesopores in K-Z5-H_2_O compared to that of K-Z5. The mesopore size distributions of K-Z5-AA and K-Z5-H_2_O&AA were similar to that of K-Z5. However, the supermicropore size of K-Z5-AA was approximately 0.91 nm, which was slightly smaller than that of K-Z5 (approximately 1.04 nm) and K-Z5-H_2_O&AA (also approximately 1.04 nm). Supermicropore size around is considered to have a strong relationship with low-pressure hysteresis loops [28]. Therefore, the size corresponding to the low-pressure loop in the nitrogen isotherms of K-Z5-AA was slightly smaller than that of K-Z5, indicating that supermicropores may have collapsed during the AA aging process.

As shown in Table 1, the total surface area, total pore volume, external surface area and micropore volume of K-Z5 were to 390 m^2^/g, 0.25 cm^3^/g, 84 m^2^/g and 0.11 cm^3^/g, respectively, suggesting the sample had abundant mesopores, consistent with the nitrogen isotherm analysis. All these features of K-Z5-H_2_O were similar to those of K-Z5, indicating almost no changes for both microstructure and supermicrostructure. After AA aging, the total surface area, external surface area, and total pore volume of K-Z5-AA decreased to 363 m^2^/g, 59 m^2^/g, and 0.22 cm^3^/g, respectively. Meanwhile, the micropore volume was 0.11 cm^3^/g, identical to that of K-Z5. The decrease in surface area was attributed to the shrinkage of the external surface due to the reaction of AA and aluminum in the framework. The surface area of K-Z5-H_2_O&AA was 381 m^2^/g, which was slightly lower than that of K-Z5 but higher than that of K-Z5-AA. Moreover, the *S_ext_* of K-Z5-H_2_O&AA was also situated between K-Z5 and K-Z5-AA. It can be considered that, due to the dilution of water, the destruction of the K-Z5 pore structure was less severe compared to treatment with pure AA.

**Figure 3:**
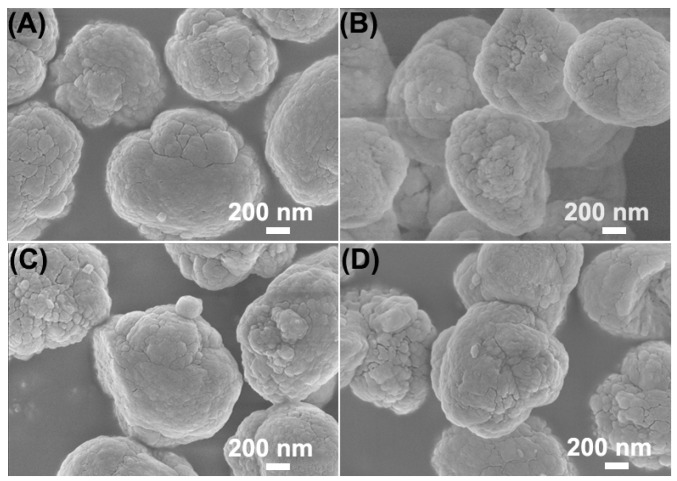
SEM images of K-Z5 (**A**), K-Z5-H_2_O (**B**), K-Z5-AA (**C**) and K-Z5-H_2_O&AA (**D**).

SEM images of K-Z5, K-Z5-H_2_O, K-Z5-AA, and K-Z5-H_2_O&AA are presented in Figure 3. The morphology of K-Z5 exhibited a spheroid-shaped agglomeration formed by multiple nanocrystals. ZSM-5 zeolite with spheroid morphology was typically obtained using TPABr as the structure-directing agent [29]. The grains of K-Z5 appeared to consist of multiple nanocrystals, with slit-shaped mesopores clearly observable on the surface. The morphology of the other three samples were similar to that of K-Z5.

**Figure 4:**
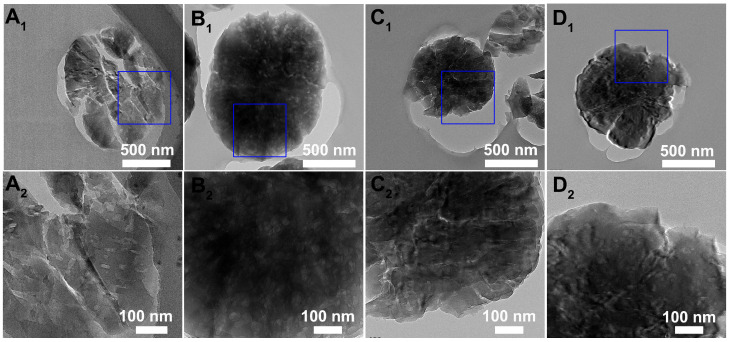
Interior cross-sectional TEM images of K-Z5 (**A_1_**,**A_2_**), K-Z5-H_2_O (**B_1_**,**B_2_**), K-Z5-AA (**C_1_**,**C_2_**) and K-Z5-H_2_O&AA (**D_1_**,**D_2_**).

Interior cross-sectional TEM images of all samples are presented in Figure 4. Smaller particles were selected to enhance the accuracy of observation, the blue squares in the low-resolution image are magnified to allow for a more detailed observation of the pore structure, and are presented through high-resolution images. The grains of K-Z5 were agglomerates composed of multiple nanocrystals, consistent with the SEM results. Abundant mesopores were observed within the crystals. The TEM image of K-Z5-H_2_O was similar to that of K-Z5, and the high-resolution image revealed distinct mesopores as those of K-Z5, indicating that no structure damage occurred after aging with water. The grains of K-Z5-AA were also agglomerations of nanocrystals; however, the mesopores in the high-resolution image were too indistinct to be clearly resolved. This indicates that some of the mesopores were impaired after aging with AA, resulting in a reduction in the external surface area. In contrast, numerous mesopores can still be observed in high-resolution TEM images of K-Z5-H_2_O&AA. Although the size appears smaller than that of K-Z5 and K-Z5-H_2_O, it indicates that some mesopores were still damaged after aging with the mixture of water and acetic acid. Nevertheless, the situation was better than that of K-Z5-AA.

**Figure 5:**
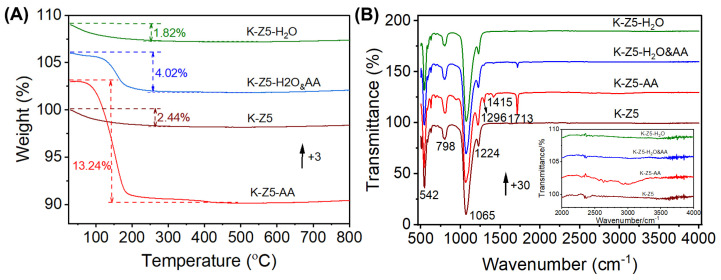
Compositional characterizations of K-Z5, K-Z5-H_2_O, K-Z5-AA and K-Z5-H_2_O&AA. (**A**) TG analysis. (**B**) IR spectra.

The adsorption capacity was determined by TG, as depicted in Figure 5A. The weight losses of K-Z5, K-Z5-H_2_O, K-Z5-AA and K-Z5-H_2_O&AA were 2.44, 1.82, 13.24 and 4.02%, respectively. Aging with water did not increase the water content of K-Z5, as evidenced by the fact that the weight loss of K-Z5-H_2_O was similar to that of K-Z5. Moreover, water appeared to inhibit K-Z5 from absorbing an excessive amount of AA, as demonstrated by the weight loss of K-Z5-H_2_O&AA being approximately one third of that of K-Z5-AA.

ATR-IR was employed for the characterization and determination of the functional groups in the samples, as shown in Figure 5B. The absorption bands at 542, 798, 1065 and 1224 cm^−1^ in the spectra of all samples pertained to the ZSM-5 zeolite [30], with no significant intensity difference in the ZSM-5 absorption bands observed among all samples. The IR spectra of K-Z5 and K-Z5-H_2_O were nearly identical, consistent with their identical weight loss behavior. In addition to the characteristic absorption bands of the zeolite, the band at 1713 cm^−1^ in the spectra of K-Z5-AA was attributed to *υ*_C=O_ of surface-adsorbed AA, while the absorption band at 1415 cm^−1^ was ascribed to *δ*_asym_ of -CH_3_ [31,32]. The absorption bands of AA were also detected in IR spectra of K-Z5-H_2_O&AA, but their intensities were significantly lower than those in K-Z5-AA. The intensities of the acetic acid absorption bands were closely correlated with the weight loss observed in the thermogravimetric analysis. Notably, the characteristic absorption band at ~3610 cm^−1^ corresponding to Brønsted acid sites—specifically associated with bridging hydroxyl groups in zeolites—was absent in the IR spectra of all four investigated samples. This observation aligns with established cation exchange mechanisms, where replacement of H^+^ by metal cations typically eliminates these acidic sites [33,34,35]. The intensity of this bridging hydroxyl vibration band serves as a quantitative indicator of cation exchange efficiency [36]. The complete absence of the 3610 cm^−1^ band in K-Z5 conclusively demonstrates full exchange of H^+^ with alkali metal ions, verifying successful cation substitution.

It is widely acknowledged that aluminosilicate zeolites, which have charge-compensating cations within their framework, show a distinct hydrophilic nature. This feature endows the zeolite with a considerable affinity for water [37,38]. The water adsorption mechanisms differ significantly between H-ZSM-5 and K-ZSM-5. In H-ZSM-5, a single water molecule coordinates with a Brønsted acid site (Si–OH–Al group), whereas multiple water molecules form stable hydration clusters around alkali metal cations (e.g., K^+^) in K-ZSM-5 [39]. The hydrothermal aging experiment may induce partial proton-driven cation exchange, leading to a depletion of framework-balanced potassium species and consequently reducing water adsorption in K-Z5-H_2_O relative to pristine K-Z5. The Brønsted acid absorption bands in the IR spectra of K-Z5-H_2_O remained undetectable, which can be ascribed to both the exceptionally low aluminum content in K-Z5-H_2_O and the limited conversion of potassium-associated framework sites (Si–OK–Al) into protonated acid sites (Si–OH–Al).

### 3.2. Acoustic Enhancement Performance

**Figure 6:**
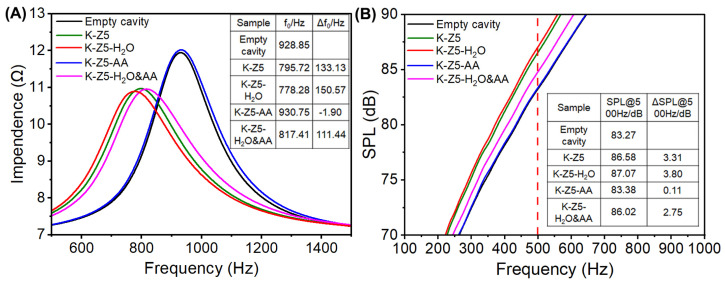
Impedance curve (**A**) and frequency response curve (**B**) of K-Z5, K-Z5-H_2_O, K-Z5-AA and K-Z5-H_2_O&AA.

The impedance and frequency response curves of K-Z5, K-Z5-H_2_O, K-Z5-AA and K-Z5-H_2_O&AA are presented in Figure 6, the red dash line in Figure 6B facilitates a direct understanding of the sound pressure levels of all samples at a frequency of 500 Hz. The resonance frequency (*f*_0_) of microspeaker was decreased from 928.85 Hz to 797.72 Hz when K-Z5 was filled into the back volume, and SPL@500Hz was increased from 83.27 dB to 86.58 dB. The offset of the resonance frequency (Δ*f*_0_) of K-Z5 was 133.13 Hz, and the increase in SPL (ΔSPL@500Hz) was 3.31 dB. A positive ΔSPL@500Hz value confirms enhanced acoustic output characteristics, demonstrating measurable improvements in the microspeaker’s performance through increased sound pressure levels. It was remarkable that K-Z5-H_2_O exhibited a superior acoustic enhancement performance, with Δ*f*_0_ being 150.57 Hz and ΔSPL@500Hz being 3.80 dB. The excessive uptake of AA did affect the acoustic enhancement performance of K-Z5, as evidenced by the Δ*f*_0_ of K-Z5-AA was −1.9 Hz and ΔSPL@500Hz was merely 0.11 dB, seemingly showing no improvement in the performance. When the AEMs in the back volume fail to effectively adsorb and desorb air molecules, the volume occupied by AEMs lowers the compliance of microspeakers, leading to an increase in *f*_0_ and a decrease in SPL@500Hz. Therefore, *f*_0_ in the presence of K-Z5-AA was nearly identical to that of the empty cavity, suggesting that K-Z5-AA did not necessarily imply ineffectiveness but rather a limitation due to its inability to adsorb and desorb air molecules effectively. The acoustic enhancement performance of K-Z5-H_2_O&AA was lower, with Δ*f*_0_ being 111.44 Hz and ΔSPL@500Hz being 2.75 dB.

When the AEMs were filled in the back volume of the microspeakers, the adsorption of air under sound pressure resulted in a reduction in the compliance of the microspeakers, causing a reduction in *f*_0_ and an increase in SPL@500Hz. Hence, the air adsorption capacity was regarded as a critical factor for AEMs [8]. The enhanced performance of K-Z5-H_2_O can be attributed to its lower weight loss, as the reduced water content enables it to exhibit a greater capacity for adsorbing air molecules. In contrast, the inferior performance of K-Z5-AA was due to its remarkably high adsorption amount of AA. The pores, particularly the mesopores observed in the TEM images, were damaged, thereby hindering the adsorption of air molecules. On the other hand, the superior performance of K-Z5-H_2_O&AA compared to K-Z5-AA could be attributed to its lower weight loss and the fact that its mesopores were less clogged than those of K-Z5-AA.

### 3.3. Adsorption–Desorption Behavior Studied by In Situ DRIFTS

**Figure 7:**
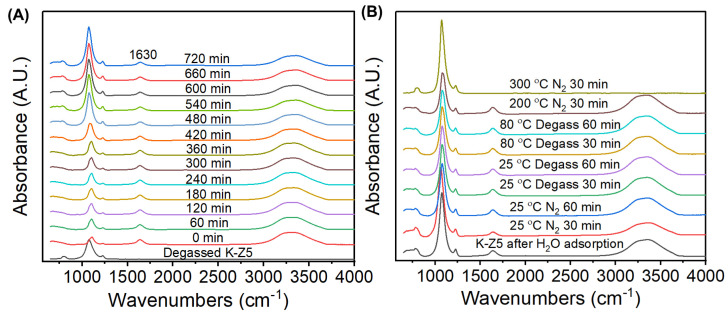
In situ DRIFTS spectra during adsorption (**A**) and desorption (**B**) of water on K-Z5.

In situ DRIFTS was utilized to explore the adsorption–desorption behavior associated with the aging characteristics of AEMs. Figure 7 displays the corresponding DRIFTS spectra for water adsorption (A) and desorption (B). The adsorption experiment was conducted at 85 °C in a controlled atmosphere consisting of nitrogen and water vapor (85% relative humidity) for 720 min under standard atmospheric pressure (101.3 kPa). Sequential desorption stages were conducted as follows: (1) nitrogen flushing at 25 °C for 60 min, (2) degassing at 25 °C for 60 min, (3) degassing at 80 °C for 60 min, (4) nitrogen flushing at 200 °C for 30 min, and (5) nitrogen flushing at 300 °C for 30 min.

It is evidenced from the spectra at 0 min in Figure 7A that water was immediately adsorbed by K-Z5. The absorption band of ZSM-5 at ~1100 cm^−1^ decreased significantly, while the intensity of water absorption bands [40] at 1630 cm^−1^ and broad band in the range of 3000~3700 cm^−1^ increased sharply. The intensity of the ZSM-5 bands increased with the extension of the adsorption time, while the water absorption bands remained stable. It should be noted that the intensity of the band at ~1100 cm^−1^, assigned to the asymmetrical stretching vibration of the internal T-O bond in the zeolite [30], increased significantly at 480 min. The migration of water from the surface to the micropore might be one reason for the increased intensity, but the intensity of the band was far beyond that of degassed K-Z5, suggesting that the structure of the zeolite was strengthened since the hydrothermal method was usually employed to anneal the silanol defect of the zeolite [41]. It has been reported that a low-temperature steam (<200 °C) treatment can heal the silanol defects and form new framework Si-O-Si bonds [42]. Therefore, it can be inferred that new T-O bonds were formed under water vapor treatment, resulting in the enhanced intensity of the band at ~1100 cm^−1^.

The desorption process of water was straightforward. A portion of the adsorbed water was flushed out by nitrogen at 25 °C, as evidenced by the decreased intensity of the water absorption bands. This suggests that the adsorption of water on ZSM-5 involves physical adsorption. Some water molecules were retained in the micropores, as indicated by the increased intensity of the water absorption bands when the environment was switched from nitrogen to vacuum. A fraction of the water was tightly bound to the zeolite framework and could not be fully removed until 300 °C. The enhancement intensity of band at ~1100 cm^−1^ did not decline, implying that the strengthened structure during the adsorption process was reliable.

The initial IR spectra (0 min of adsorption) indicate the immediate hydration of K-Z5, thereby confirming its hydrated state under operational conditions. Nitrogen isotherms of non-degassed hydrated samples (K-Z5 and K-Z5-H_2_O) were acquired without prior thermal pretreatment (see Figure S5). The most significant difference between the two isotherms is the low-pressure hysteresis loop observed in *P/P*_0_ = 0.1~0.4, which is associated with defects in the zeolite structure [43]. Compared to nitrogen isotherms obtained after degassing pretreatment (Figure 2B), the low-pressure hysteresis loop disappeared in K-Z5 in its hydrated state but reappeared in the nitrogen isotherm of K-Z5-H_2_O in its hydrated state. Since defects are related to terminal silanol [44] with high hydrophilicity [45], it can be inferred that terminal silanol was healed during the hydrothermal treatment, leading to reduced water adsorption.

The reduced weight loss observed in K-Z5-H_2_O relative to pristine K-Z5 can be primarily attributed to (1) a portion of the potassium cations being exchanged with protons, and (2) structural healing of silanol defects. The lower water content enables K-Z5-H_2_O to exhibit a better performance.

**Figure 8:**
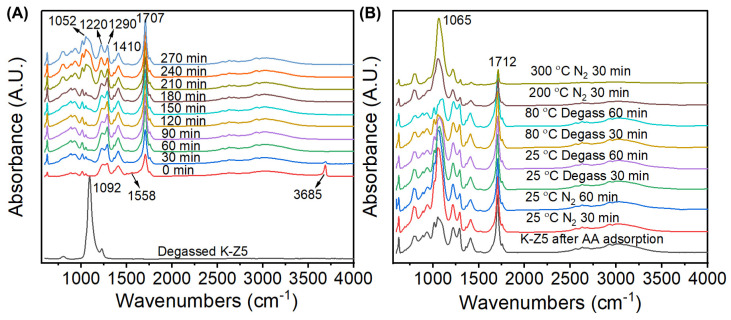
In situ DRIFTS spectra during adsorption (**A**) and desorption (**B**) of AA vapor on K-Z5.

The in situ DRIFTS spectra of the adsorption and desorption of AA on K-Z5 are presented in Figure 8. The adsorption process of AA was conducted at 85 °C and the adsorption time was 270 min. The absorption band of K-Z5 at 1092 cm^−1^ attributed to the asymmetrical stretching vibration of the internal T-O bond completely disappeared at 0 min, indicating that AA adsorption on K-Z5 occurred rapidly with a significant adsorption capacity, as if the zeolite framework was fully saturated by AA. The bands observed at 1410, 1707 and 2500~3500 cm^−1^, which are characteristic of AA, suggest that the adsorption of AA on K-Z5 was mainly molecular adsorption.

In addition to the bands characteristic of AA, a weak but distinct band at 1558 cm^−1^ was detected, which was assigned to the υ_asymCOO−_ band of acetate ion in zeolite [46], assuming that potassium acetate was generated due to the reaction of AA and Si-O(K)-Al in the zeolite framework. The potassium ions were exchanged for protons and formed Brønsted acid sites. An initial dehydration step was thought to occur during the adsorption of AA on HZSM-5, resulting in the formation of an acyl intermediate, water, carbon dioxide, etc. [47]. The generation of water and carbon dioxide was identified by MS spectra (Figure S2) during the desorption process of AA in TPD test. Species of water (m/s = 18) and carbon dioxide (m/s = 44) were observed under 100 °C. The water generated was adsorbed by Brønsted acid site immediately, with clear evidence that a sharp band at 3685 cm^−1^ assigned to unperturbed -OH stretching vibration of the H_3_O^+^ was observed in the adsorption spectra at 0 min [39]. The proposed reaction of acetic acid and K-Z5 can be found in Figure S3. The characteristic absorption bands at 1558 cm^−1^ and 3685 cm^−1^ exhibited progressive attenuation with prolonged adsorption duration, showing complete signal extinction after 60 min. It was assumed that they were covered by the continuous adsorption of AA, as verified by the increased intensity of the band at 1707 cm^−1^. Notably, the framework vibration signatures of zeolite (800 cm^−1^ symmetric stretching and 1092 cm^−1^ asymmetric stretching of T-O-T bonds) remained undetectable until 210 min, suggesting adsorbed AA molecules gradually diffused into the micropores.

Desorption of AA was performed in the same sequence under different conditions (Figure 8B). The significant enhancement in the ZSM-5 zeolite characteristic bands at 800 and 1065 cm^−1^ after nitrogen flushing at 25 °C for 30 min indicated that the adsorption of AA involves a portion of physical adsorption. The absorption band of zeolite at 1065 cm^−1^ weakened the following gas phase transition from nitrogen atmosphere to vacuum conditions, suggesting that some AA molecules were adsorbed in the micropores and extracted outside by degassing. There was a very small amount of AA that binds very strongly to the zeolite, as evidenced by the bands of AA observed when the temperature increased to 200 °C. The broad bands in the range of 2500~3500 cm^−1^ disappeared at 300 °C, but the band at 1712 cm^−1^ was still notable, combined with the bands at 1430 cm^−1^ and 1290 cm^−1^ assigned to *γ*_C-O_ and *δ*_asymCH3_, respectively, suggesting that acyl intermediate still persisted within the zeolite. The MS spectra (Figure S2) further indicated that the desorption of AA from the zeolite was complicated. The reaction generated water, carbon dioxide, and other species which were difficult to distinguish, and the desorption process persisted until 500 °C.

According to the DRIFTS spectra and TEM results, AA was adsorbed both in the mesopores and micropores of zeolite. The adsorption process entailed the reaction between AA and zeolite. The absorption bands of ZSM-5 were not discernible until 210 min, suggesting that K-Z5 adsorbed a considerable quantity of AA, and the desorption of AA was arduous, which resulted in a poor acoustic enhancement performance.

**Figure 9:**
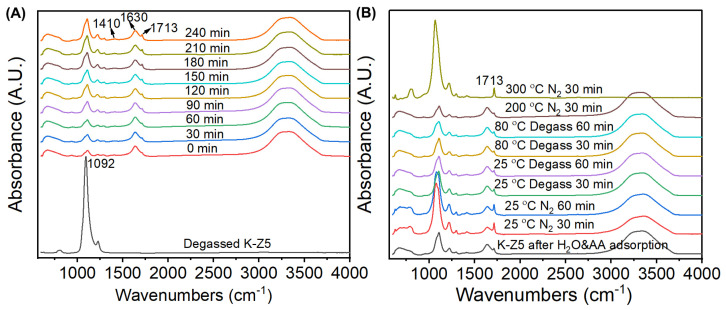
In situ DRIFTS spectra during adsorption (**A**) and desorption (**B**) of AA&H_2_O vapor on K-Z5.

The in situ DRIFTS spectra of adsorption and desorption of AA&H_2_O vapor on K-Z5 are presented in Figure 9. No bands corresponding to acetate and hydronium ions were observed throughout the entire adsorption process, suggesting that water molecules were initially adsorbed by Si-O(K)-Al sites and no reaction occurred between AA and zeolite. The adsorption bands of ZSM-5 zeolite, which completely disappeared during AA adsorption, could still be observed during the adsorption process, suggesting a relatively small adsorption amount compared to AA adsorption. The intensity of the band at 1713 cm^−1^ was very weak compared to the adsorption band at 1630 cm^−1^ and the wide band in the range of 3000~3700 cm^−1^, which are characteristic of water, further suggesting that water was adsorbed by zeolite prior to AA.

The DRIFTS spectra (Figure 9B) during the desorption process of AA aqueous solution were analogous to those of AA. The physically adsorbed water was effectively removed by nitrogen purging at 25 °C, as evidenced by the significantly enhanced intensity of the band at ~1100 cm^−1^. The intensity of the band weakened following the gas phase transition from a nitrogen atmosphere to vacuum conditions as a result of the extraction of water from micropores. It should be noted that there was still some water and AA that did not desorb at 200 °C. The absorption bands of water completely disappeared at 300 °C, but the bands at 1410 and 1713 cm^−1^ persisted, supposing the two bands that belonged to acyl intermediates that were generated by the reaction of AA and zeolite remain stable within the framework. Thus, the better acoustic enhancement performance of K-Z5-H_2_O&AA compared to K-Z5-AA can be explained. Water effectively protected K-Z5 from adsorbing an excessive amount of AA, and most of the adsorbed water can be easily removed compared to the sample that adsorbed AA.

## 4. Conclusions

This study systematically investigated the aging characteristics of AEMs with the K-type ZSM-5 zeolite. The pores are of great significance for zeolite-based AEMs, and the blockage of pores would lead to the performance degradation. The aging behavior of AEMs is intricate and multifaceted. Especially, the adsorption–desorption behavior of various adsorbates, such as moisture and AA, hold substantial significance. The adsorption of AA involves both physical and chemical adsorption, along with the generation of water, carbon dioxide, etc., resulting in an excessive adsorption quantity of AA and causing severe degradation of acoustic enhancement performance; the offset of the resonance frequency was decreased from 133.13 Hz to −1.90 Hz and the increase in sound pressure level was reduced from 3.31 dB to 0.11 dB. However, water vapor, considered to be a critical factor in the aging characteristics of AEMs, is not detrimental. The performance of the sample aged with water was improved due to the strengthening of the zeolite structure; the offset of the resonance frequency was increased to150.57 Hz and the increase in the sound pressure level was elevated to 3.80 dB. Moreover, when the zeolite sample was aged with the AA aqueous solution, water effectively prevented the sample from adsorbing excessive AA, preserving most of the acoustic enhancement performance; the offset of the resonance frequency was 111.44 Hz and the increase in the sound pressure level was 2.75 dB. Our comparative analysis of zeolite-based AEMs demonstrates that acidic gaseous species constitute the dominant degradation mechanism, whereas water vapor forms a protective molecular barrier against excessive acid adsorption through competitive absorption effects. We anticipate that this paper will contribute to the development of superior anti-aging AEMs.

## Figures and Tables

**Table 1:** SAR and textual properties of K-Z5, K-Z5-H_2_O, K-Z5-AA and K-Z5-H_2_O&AA.

Sample	*SAR*	*S*_total_/(m^2^·g^−1^)	*V*_total_/(cm^3^·g^−1^)	*S*_ext_/(m^2^·g^−1^)	*V*_micro_/(cm^3^·g^−1^)
K-Z5	608	390	0.25	84	0.11
K-Z5-H_2_O	613	392	0.25	84	0.10
K-Z5-AA	646	363	0.22	59	0.11
K-Z5-H_2_O&AA	617	381	0.24	68	0.13

## Data Availability

Data are contained within this article.

## Supplementary Materials

The following supporting information can be downloaded at: https://www.mdpi.com/article/10.3390/nano15090639/s1, Figure S1: Illustration of storage with acetic acid or acetic acid aqueous solution; Figure S2. Mass spectra during the desorption process following acetic acid adsorption in TPD; Figure S3. Proposed mechanism of acetic acid ketonization on K-Z5. Figure S4. Pore structure of ZSM-5 zeolite [48]; Figure S5. Nitrogen isotherms without pretreatment of K-Z5 and K-Z5-H_2_O.
