# Supplementary material for: Aging Characteristics of ZSM-5 Zeolite on Low-Frequency Acoustic Applications
Source: Nanomaterials (Basel). 2025 Apr 23;15(9):639. doi: 10.3390/nano15090639 (PMC12073534; doi:10.3390/nano15090639)
Supplement: Supplementary file 1 [file nanomaterials-15-00639-s001.zip › nanomaterials-3575490-supplementary.pdf]

Supplementary material

Aging Characteristics of ZSM-5 Zeolite on Low-Frequency Acoustic Applications

Mingbo Guo[1,2], Yijun Wang[2], Lei Zhang[2], Junran Lu[2], Chang Gong[2], Wanning Zhang[1], Yuxi Fang[1], Xinyuan Zhu[1*], Shunai Che[1*]

1. State Key Laboratory of Synergistic Chem-Bio Synthesis, School of Chemistry and Chemical Engineering, Frontiers Science Center for Transformative Molecules, Shanghai Key Laboratory for Molecular Engineering of Chiral Drugs, Shanghai Jiao Tong University, Shanghai 200240 P. R. China

2. SSI New Material (Zhenjiang) Co.,Ltd., 7 Songlin Mountain Road, Zhenjiang, 212006 P. R. China

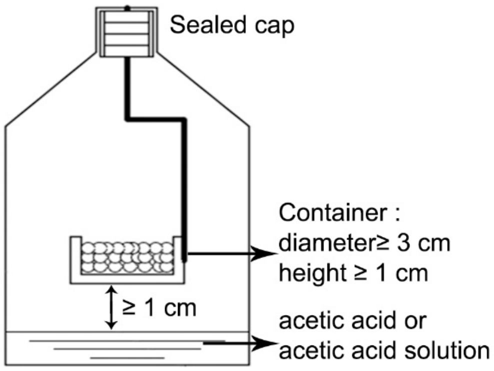

Figure S1. Illustration of storage with acetic acid or acetic acid aqueous solution

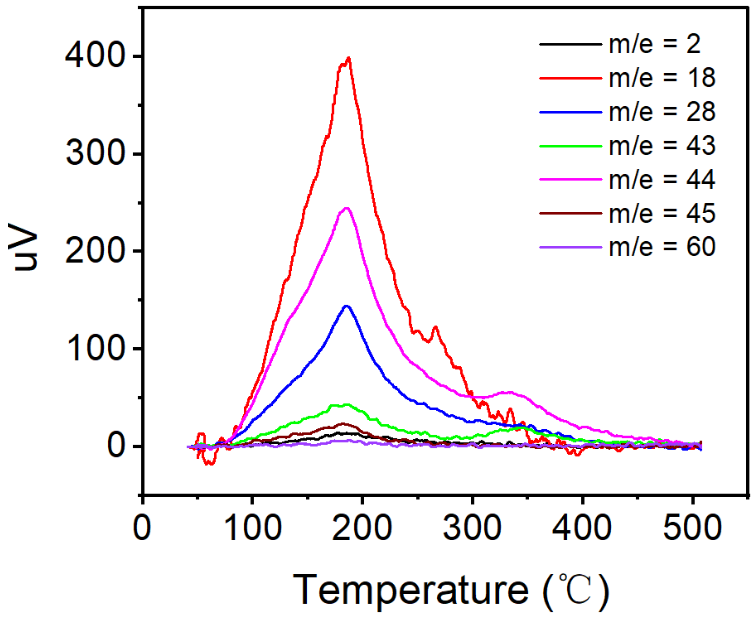

Figure S2. Mass spectra during the desorption process following acetic acid adsorption in

TPD

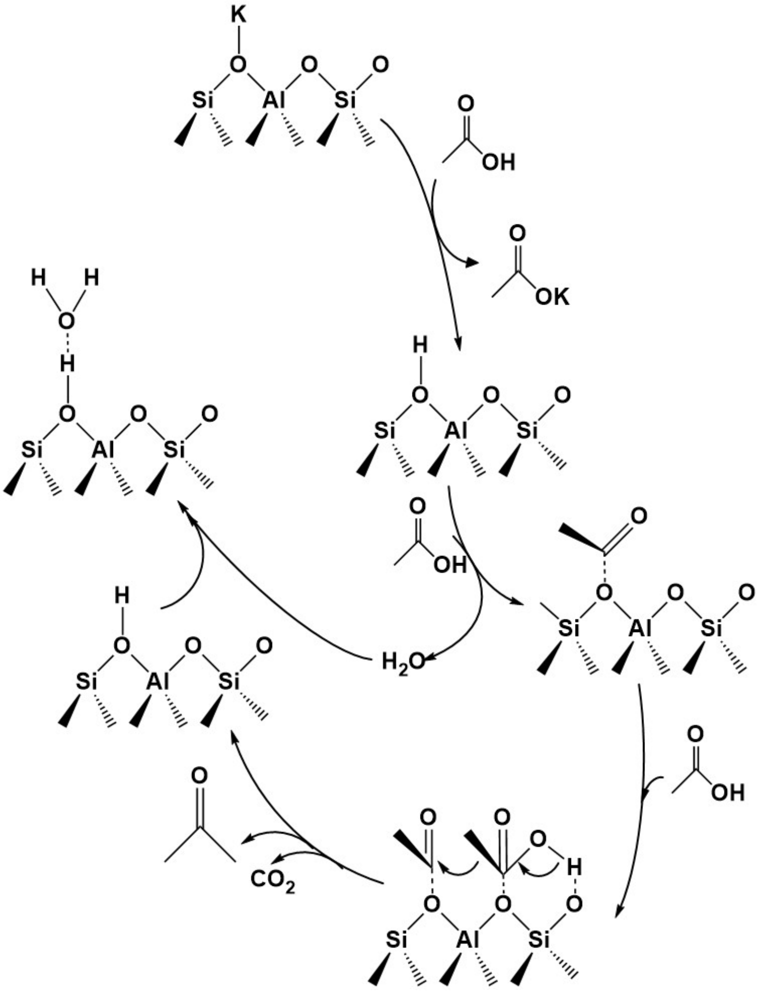

Figure S3. Proposed mechanism of acetic acid ketonization on K-Z5

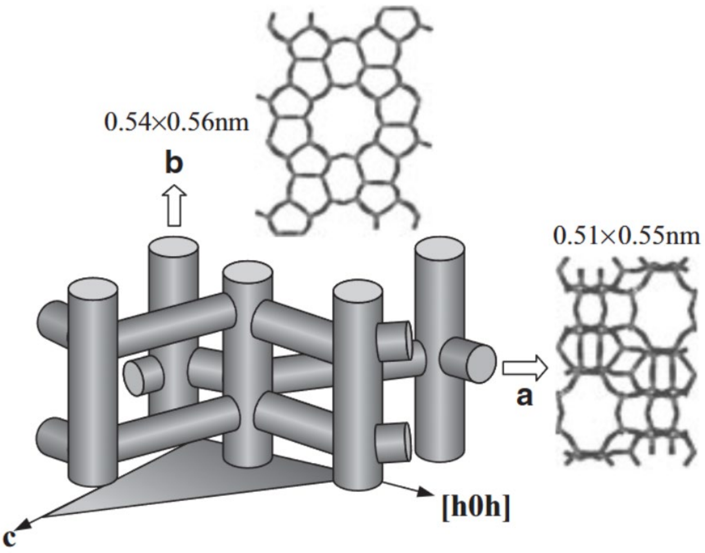

Figure S4. Pore structure of ZSM-5 zeolite[1]

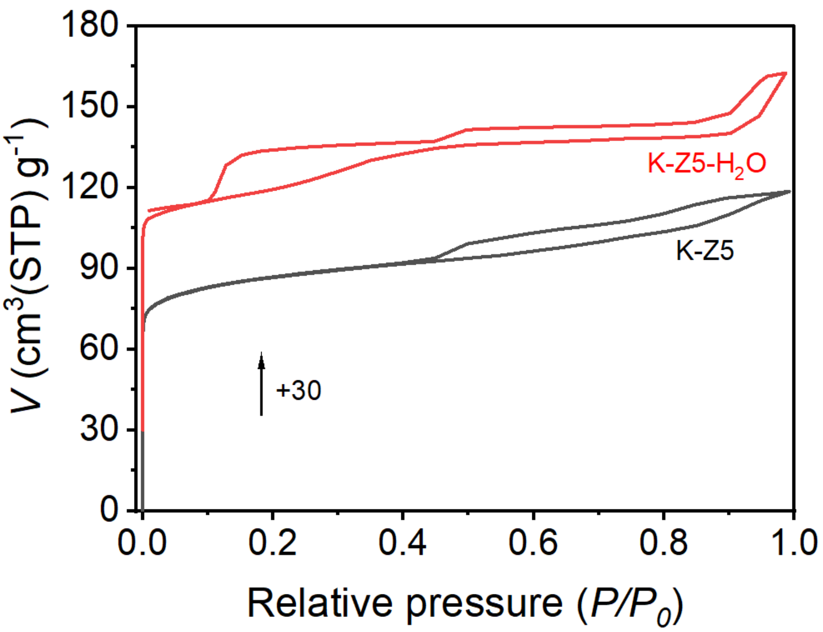

Figure S5. Nitrogen isotherms without pretreatment of K-Z5 and K-Z5-H_2O

[1] Lai, Z., Tsapatsis, M., and Nicolich, J. P., Siliceous ZSM-5 Membranes by Secondary Growth of b-Oriented Seed Layers. Advanced Functional Materials **2004**, 14, 716-729. https://doi.org/10.1002/adfm.200400040.
